# MicroCT X-ray comparison of aligner gap and thickness of six brands of aligners: an in-vitro study

**DOI:** 10.1186/s40510-020-00312-w

**Published:** 2020-05-11

**Authors:** Luca Lombardo, Mario Palone, Mattia Longo, Niki Arveda, Michele Nacucchi, Fabio De Pascalis, Giorgio Alfredo Spedicato, Giuseppe Siciliani

**Affiliations:** 1 Postgraduate School of Orthodontics, University of Ferrara, Via Luigi Borsari 46, 44121 Ferrara, Italy; 2 Division for Sustainable Materials, Research Centre of Brindisi, S.S. 7 Appia km 706,00, I-72100 Brindisi, Italy; 3 School of Economics, Management and Statistics, University of Bologna, Bologna, Italy

**Keywords:** Clear aligner therapy, Micro computed tomography, Aligner thickness, Aligner gap

## Abstract

**Background:**

To investigate and compare the gap (i.e. fit) and thickness of six aligner systems (Airnivol, ALL IN, Arc Angel, F22, Invisalign and Nuvola) using industrial computed tomography (CT). The null hypothesis was that there would be no detectable differences in either measurement between the aligners investigated.

**Materials and methods:**

Passive aligners of each brand were fitted to one single resin cast prototyped from an STL file from a single patient. The samples obtained were examined under high-resolution micro-CT, and the resulting tomographic microphotographs and volumetric data were compared. 3D analysis investigated the gap volume, the mean gap width and the maximum gap width of each sample. A total of 204 linear 2D measurements were made on 18 microtomographic images to investigate the aligner gap and thickness among different systems. Investigated regions were the central incisor, canine and first molar. The resulting measurements were analysed by ANOVA and compared using Tukey’s post hoc analysis (*P* < 0.05).

**Results:**

3D analysis revealed that the F22 displayed lower gap volume and mean gap width, followed by Airnivol and Invisalign, whereas Airnivol the lowest maximum gap width. 2D analysis showed that F22 had the lowest mean gap and aligner thickness at all teeth investigated. Comparison of the 2D point values revealed statistically significant differences between brands in terms of both measurements (*P* < 0.05), with the exception of six points in the gap analysis and one in the thickness analysis.

**Conclusions:**

There are differences between the six aligner systems examined in terms of 2D and 3D measurements of aligner thickness and gap.

## Introduction

With the introduction of Computer-Aided Design and Computer-Aided Manufacturing (CAD/CAM) technology to Orthodontics, Align Technology (Santa Clara, CA, USA) launched its first clear orthodontic aligner in 1998 [1]. The demand for such orthodontic devices has grown, and they now occupy a significant portion of the market as a valid alternative to traditional fixed appliances. Indeed, the rise in popularity of clear aligner therapy (CAT) has been fuelled by the increasing demand of adult patients for more aesthetic treatments that do not negatively affect their social lives or relationships [2], and that are associated with fewer periodontal complications and a lower risk of root resorption [3].

Initially, CAT was mainly indicated in simple non-extraction cases, but over the years they have evolved, and there is now good evidence of their efficacy and efficiency [4, 5]. Nowadays, they are a therapeutic option even in complex cases that involve distalization and space closure movements [6].

As regards the analysis of single movements, the literature agrees that rotation of cone-shaped teeth [4] and movements that require good root control [6] are those most difficult to obtain with aligners, while crown tipping and intrusion are the most predictable [7–9]. In contrast, the literature contains differing reports on the efficacy of aligners in achieving extrusion [10]. That being said, it is generally agreed that aligners are particularly efficient at resolving malocclusions of slight to moderate complexity in non-extraction cases due to their good capacity to expand, align and level the arches [5, 11].

There are many factors that influence the predictability of CAT, including the characteristics of the set-up (the staging of tooth movements and the types of programmed movement) [7], the use of grip points and auxiliaries such as elastics and buttons [12], aligners’ physical properties, the manufacturing method of the thermoplastic materials used to make them [13], and the extension of their gingival margins [14].

The thickness of the aligner material can affect not only their optical properties [15], but also the forces and moments expressed by the device [16], which often exceed those considered as optimal in the literature [17]. In fact, it has been reported that acceptable forces for tipping (0.5–0.75 N) and intrusion (0.1–0.25 N) may be exceeded by as much as tenfold [18]. For these reasons, Kwon et al. advise keeping programmed movements within the range 0.2–0.5 mm [19], and Elkholy et al. suggest using aligners of nominal thickness 0.4 mm in order to minimise the initial overload on the periodontal tissues typical of the early stages of CAT [20].

Another factor that may influence the predictability of clinical outcomes with aligners is the fit, i.e. the gap between the inner surface of aligners and the external surface of the tooth. The fit is determined by the thermoforming process (pressure and temperature), the elastic modulus of the materials used [21], the presence of divots or attachments [22] and the hygroscopic expansion that occurs in contact with saliva [23].

The only studies focusing on the topic have been carried out using a scanning electron microscope (SEM) to obtain microphotography of buccolingual sections of passive aligners, created by a cutting machine and fitted to stereolithographic models [21, 22]. The first of these studies concluded that both Invisalign (Align Technology, San Jose, CA, USA) and CA-Clear Aligner (Scheu-Dental, Iserlohn, Germany) systems provided good fit. The second study, which looked at three aligner systems, Invisalign, CA-Clear Aligner and F22 (Sweden&Martina, Due Carrare, Italy), investigated aligner fit on attachments created with composites of different viscosity in both arches; differences between the aligner systems were found, but in general a better fit was detected when high-viscosity resin was used to create the attachments, due to a lesser degree of contraction during the curing process with respect to its flowable counterparts.

Although originally, these studies did present some limitations, in particular regarding methodology and the low number of aligner brands taken into consideration. Indeed, the aligner brands studied thus far have been few considering the wide range of aligner systems on the market [24], and relying on a cutting machine with continuous irrigation to obtain the aligner sections presents a risk, albeit minimal, of imprecisions in the edges due to overheating that might cause smearing.

In order to overcome these limitations, we set out to investigate both the gap and thickness of six brands of aligner using a non-invasive method of industrial microtomography and conduct both 3D and 2D comparative analyses on the resulting data. This was accomplished in order to test the null hypothesis that there would be no differences between the six brands of aligners investigated in terms of either aligner gap or thickness.

## Materials and methods

The design of this in vitro study had been approved by the Ethical Committee of the Postgraduate School in Orthodontics of the University of Ferrara with the registration number 6/2017 and was conducted in conformity to the Declaration of Helsinki. One set of PVS impressions (Elite HD+ Regular and Light Body, Badia Polesine, Rovigo, Italy) was obtained from a patient with skeletal Class I and minimal crowding in both arches. There were no caries, gingival recession, cervical lesions, prostheses or teeth with reduced clinical crowns. These impressions, together with the patient’s clinical records (diagnostic radiographic and photographic images), was sent to Align Technology (Santa Clara, CA, USA), as that manufacturer only accepts PVS impressions or STL files generated by I-Tero® Element^TM^ scanner (Align Technology, Santa Clara, CA, USA). After this, the corresponding STL (Stereo Lithography interface format) file was obtained from the ClinCheck online platform (Align Technology, Santa Clara, CA, USA).

Then, the STL file, together with the patient’s complete documentation, was sent to the other aligner manufacturers (Table 1). The same clinician, expert in CAT, expressed the same therapeutic aims in all cases. After approving the respective treatment plans, the explicit request was to obtain a perfectly passive upper aligner with no attachments or divots.

**Table 1:** List of six commercial aligner systems investigated, with the respective thicknesses and construction materials

Aligner	Material	Thickness (mm)	Manufacturer
Airnivol	Polyethylene terephthalate glycol (PET-G)	0.75	Airnivol srl, Navacchio di Cascina, PI, Italy
ALL IN	Polyethylene terephthalate glycol (PET-G)	0.80	Micerium, Avegno, GE, Italy
Arc Angel	Polyethylene terephthalate glycol (PET-G)	0.75	Gruppo Dextra, Modena, MO, Italy
F22	F22 Polyurethane	0.75	Sweden-Martina, Due Carrare, PD, Italy
Invisalign	SmartTrack: multi-layer aromatic thermoplastic polyurethane	0.75	Align Technology, Santa Clara, CA, USA
Nuvola	Polyethylene terephthalate glycol (PET-G)	0.75	GEO srl, Rome, RM, Italy

Meanwhile, a single resin cast was obtained from the same STL file (E-Dentstone M; EnvisionTEC, Gladbeck, DEU) using an ULTRA 3SP Ortho 3D Dental Printer (EnvisionTEC, Gladbeck, DEU), with a printing resolution set at 50 μm^3^. Before printing, the 3D printer calibration was checked in order to ascertain that the resin cast would not suffer from any deformation. We 3D printed a single resin model rather than multiple resin models for each specimen due to fact that different resin casts, even obtained using the same 3D printer at the same time, could have some micrometric variation. After washing the cast, each passive aligner was mounted on it in turn and then kept in place to avoid distortion until an X-ray investigation had been performed for each specimen, which was then removed.

Each of the six samples thereby obtained was then scanned consecutively using a nano-CT GE Phoenix Nanotom (GE Sensing & Inspection Technologies GmbH, Wunstorf, Germany), at the ENEA research centre of Brindisi (Italy). This system is equipped with a 180 kV/15 W nano-focus tube and a high-precision and extremely stable system for positioning samples with a 2300 x 2300-pixel 2D detector (Hamamatsu Photonics, Hamamatsu City, Shizuoka, Japan). The beam voltage was set at 80 kV, amperage at 180 μA and the voxel size was 15.8 μm. This machine, which features a granite base and air-cushioned turntable to minimise friction, enables the acquisition of a series of X-rays as the sample revolves 360°. The resulting data are used to create a digital volumetric reconstruction via a calculation algorithm.

These images were used to create a 3D rendering of the patient’s dentition using Avizo Fire software, Edition 8 (Thermo Fisher Scientific FEI, Hillsboro, OR, USA), installed on the nanotomograph workstation. Given the size of the volumetric files, the renderings were divided into two equal parts corresponding to the right and left sides of the arch. The dimensions of the processed datasets were equivalent to a 1996 × 3623 × 1212-voxel matrix, with a dynamic range of 8 bits per voxel (256-level grey scale), i.e. roughly 9 GB per file.

The data, images, and results of the following analyses refer to the left side of the arch.

### 3D analysis

**Fig. 1:**
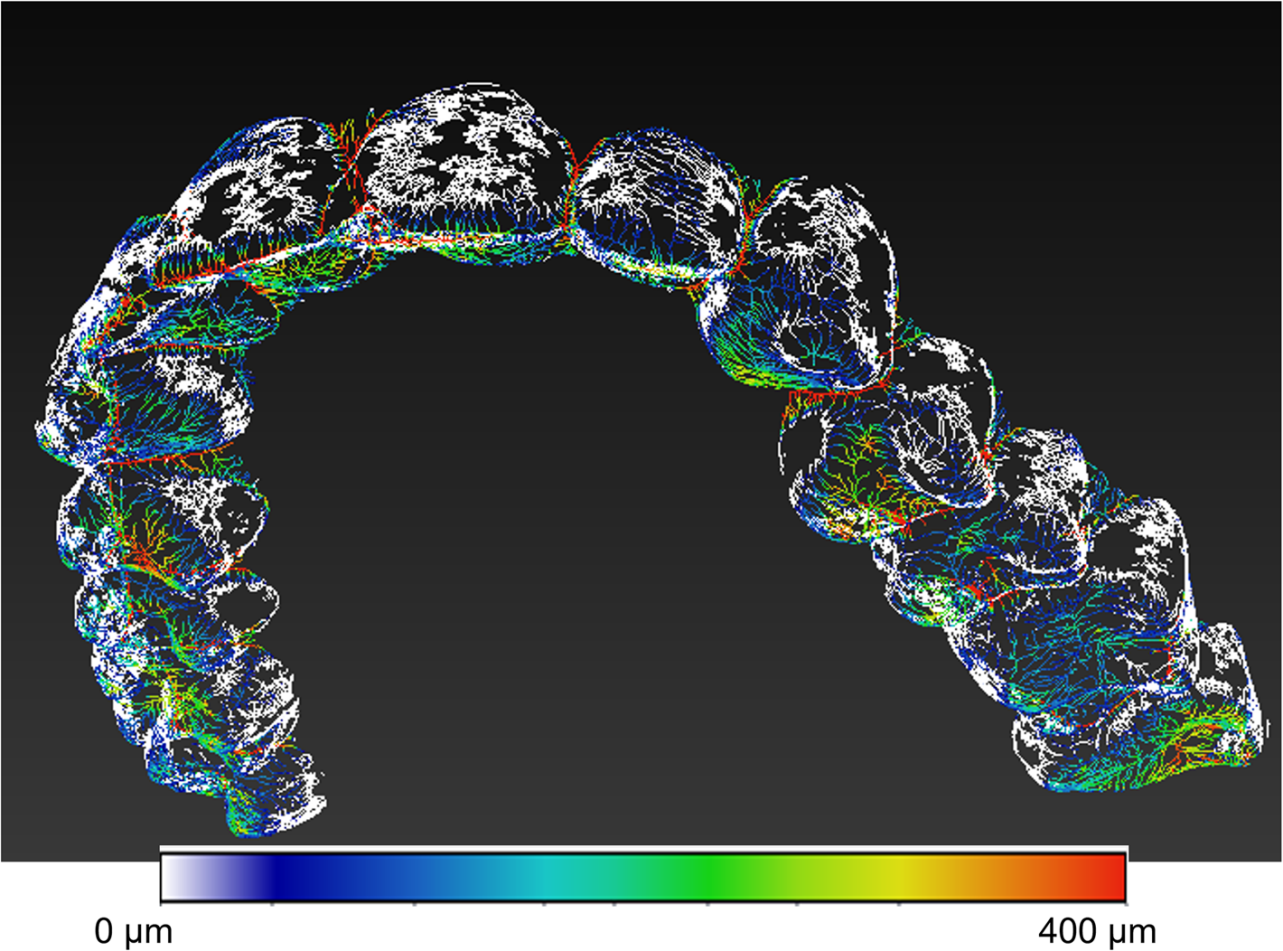
Skeleton colourimetric mapping of the distances measured. White dots indicate a minimum distance of 15 μm, and red dots greater than 400 μm

The procedure used to calculate the mean gap (mean volume, mean gap and maximum gap) was based on an algorithm proposed by Hildebran and Ruesgegger [25]. The Auto Skeleton mode of the 3D Avizo Fire processing software (Thermo Fisher Scientific FEI, Hillsboro, OR, USA) also provided a graphical map of the gap (Fig. 1). The 3D Avizo Fire software extracts, from binary image data, the medial axis of the interconnected regions, and, in essence, calculates a map of the distances in the binary images, performing an iterative narrowing of the volumes in the image in order to obtain a line of connected voxels. Each point in the skeleton (the median line), thereby preserves the information regarding the gap width, while the spatial region that was used to map the distance is the volume of the gap.

The red hues correspond to distances from the edge, i.e. thicknesses, greater than 400 μm, while the blue hues correspond to areas in which the distance is minimal.

In order to obtain uniform results, bearing in mind that each aligner brand presented different marginal finishing, gap values were assessed occlusally with respect to a plane passing through the points A, B and C.

**Fig. 2:**
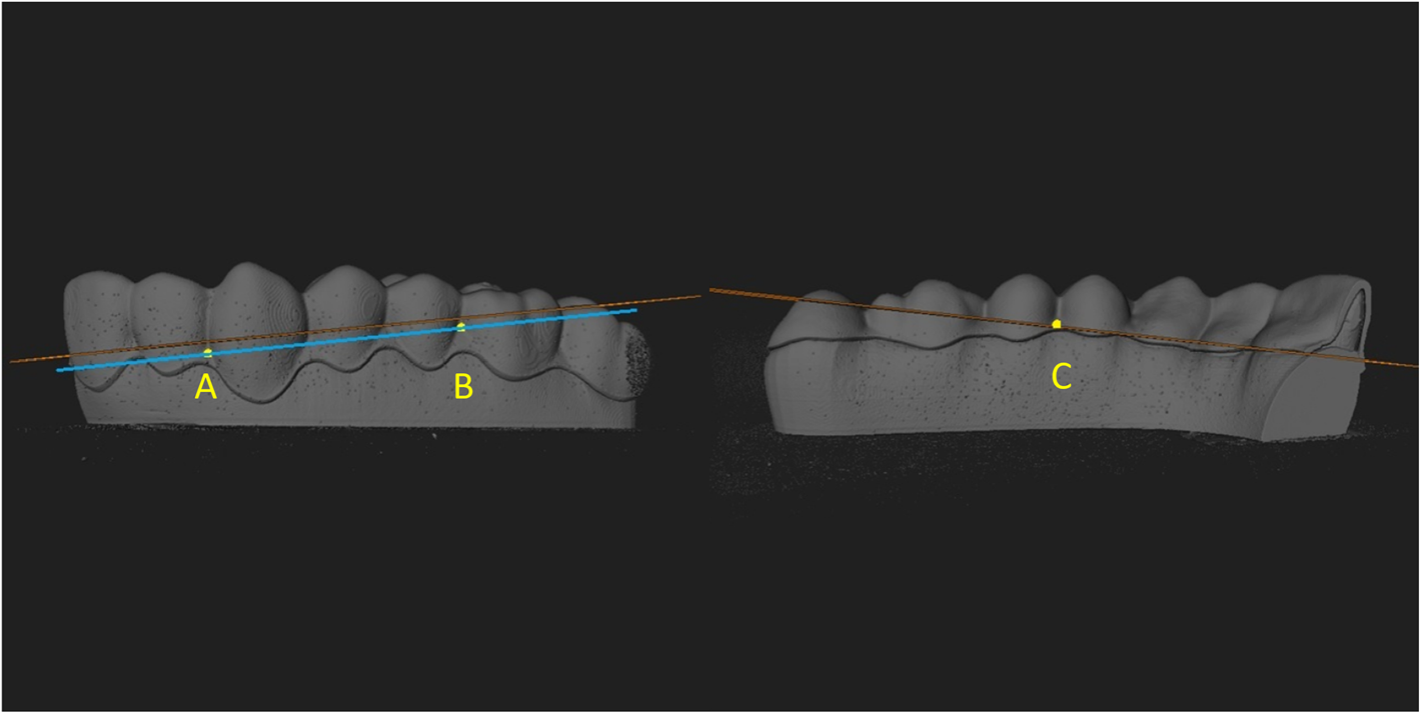
Slice passing through the interdental labial papilla between lateral incisor and canine (point A), labial papilla between first molar and second premolar (point B), and palatal papilla between premolars (point C)

The slices obtained passing through these points are shown in Fig. 2.

### 2D analysis

**Fig. 3:**
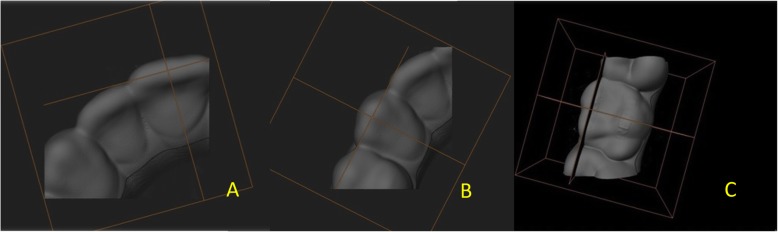
Identification of slice plane for incisor (**a**), canine (**b**) and first molar (**c**)

**Fig. 4:**
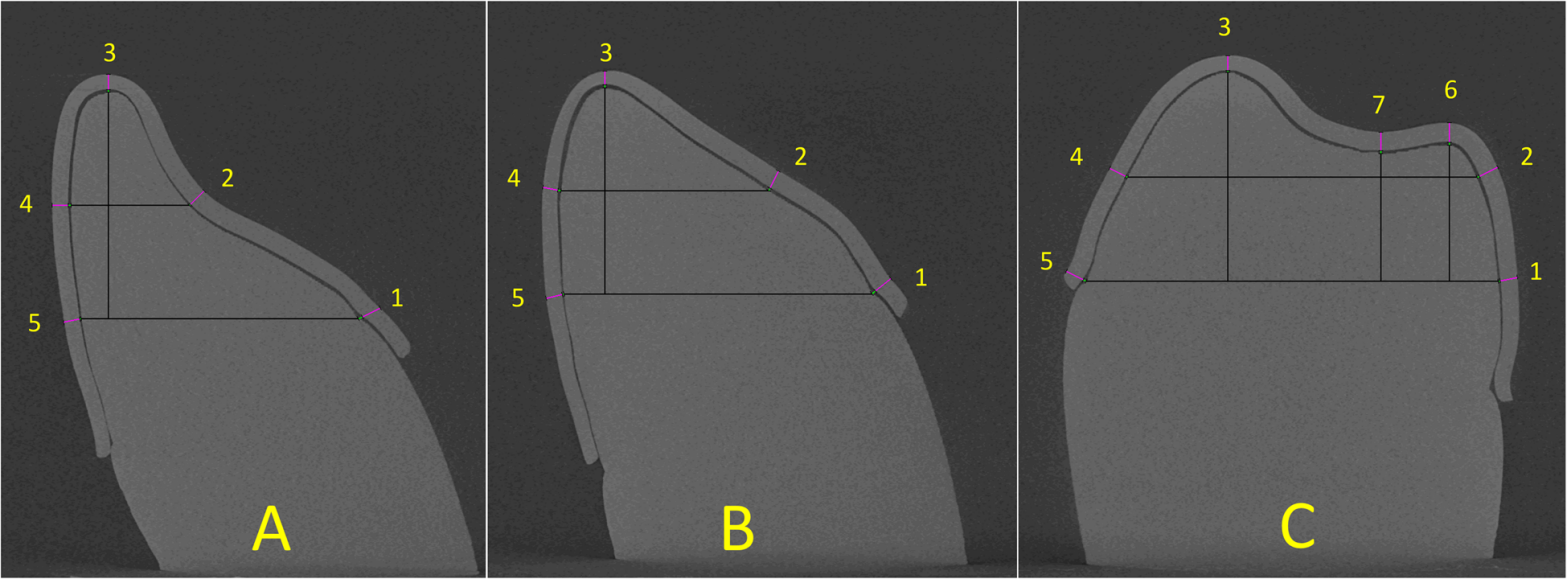
Identification of 2D points on construction grid for incisor (**a**), canine (**b**) and first molar (**c**). Eight points were identified: 1, palatal gingival; 2, midway point between 1 and 3; 3, incisal edge; 4, midway point between 3 and 5; 5, vestibular gingival; 6, vestibular cusp; 7, palatal cusp; and 8, fossa

In order to measure the aligner gap and thickness, microtomographic photographs were obtained from a total of 18 slices virtual slices corresponding to the investigated teeth (central incisor, canine and first molar. The slices were made on a plane constructed perpendicular to the axis linking the most mesial and distal points of each tooth examined and passing through its midway point (Fig. 3). This was achieved using the orientation system, which enables orientation along the spatial planes according to known angles and, exploiting the equivalence between the casts and the software capacity, positioning of the scanning slice on the same plane for all samples. On the microphotographs thereby obtained, specific grids were constructed using ImageJ software (NIH ImageJ Software, https://imagej.nih.gov/ij/), an open-source programme for analysing images for scientific purposes, on which 2D points were identified (Fig. 4). Both measures were investigated by tracing a line perpendicular to the tooth line tangent to each of these points, at × 3200 magnification. Thus, 34 measurements were performed per sample, and these measurements were repeated four times, giving a total of 1020 measurements.

### Statistical analysis

The statistical analysis enabled comparison of each system, considering both gap and aligner thickness, based on the following variables:

- Type of tooth examined (central incisor, canine or first molar)
- Point identified on the 2D grid for each microtomographic image.

The data from the 3D analysis are expressed in terms of total gap volume (mm^3^), and mean and minimum gap thickness (μm), whereas the data from the 2D analysis of the 1020 linear measurements are expressed as means (μm) ± standard deviations (SD). All analyses have been split by measure. Statistical analyses were based on two-way ANOVA and post hoc measures. Significant *P* values indicate that at least one group is different from the overall mean. Tukey’s post hoc analysis comparison of means indicates which pairs of measures are statistically different. R-Statistical software was used to perform statistical analysis. Statistical significance was assessed using a 5% threshold.

## Results

### 3D analysis

**Fig. 5:**
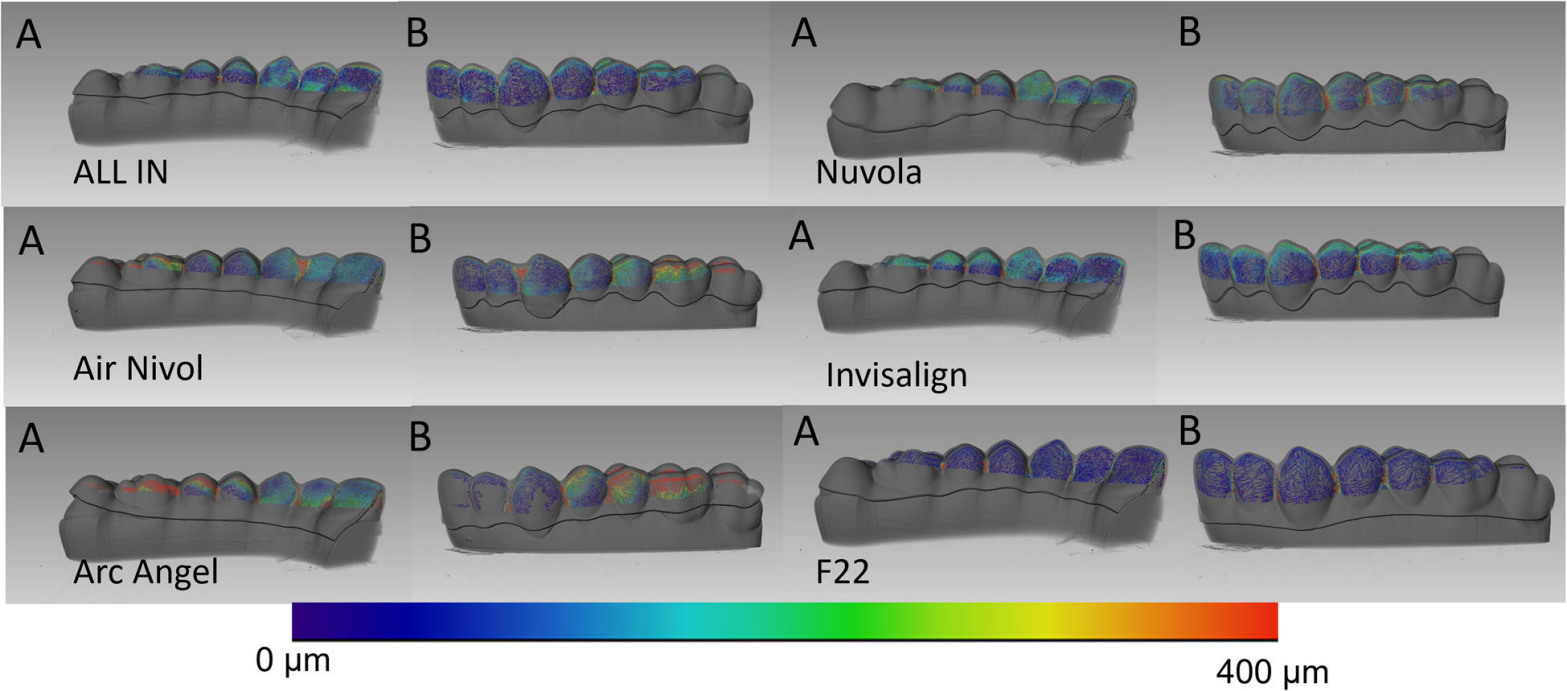
Representative colourimetric skeleton with corresponding map of the distances measured for the six aligners from internal (**a**) and external (**b**) views

**Fig. 6:**
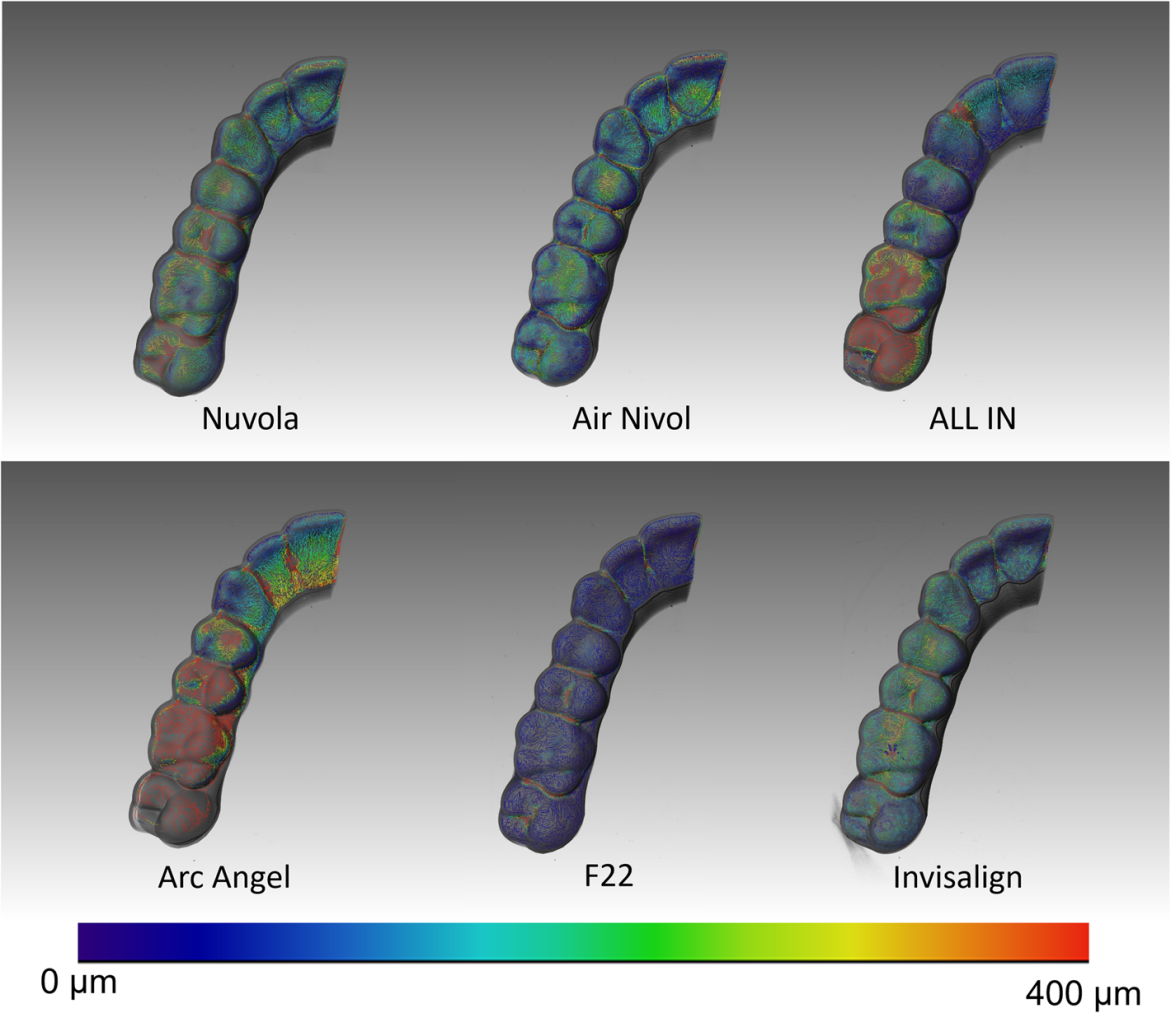
Representative colourimetric skeleton with corresponding map of the distances measured for the six aligners from occlusal view

F22 had the smallest aligner gap volume (106.7 mm^3^) and mean gap width (224.7μm), followed by Air Nivol (160.2 mm^3^, 250.8 μm) and then Invisalign (180.6 mm^3^, 269.23 μm). The smallest maximum aligner gap width value was found for Air Nivol (763.65 μm), followed by F22 (857.28 μm) and then Invisalign (915.86 μm). Results are shown in Table 2, and graphical representations of the colourimetric maps are shown from lateral (Fig. 5) and occlusal views (Fig. 6).

**Table 2:** 3D analysis of each system

	Gap volume (mm^3^)	Mean gap width (μm)	Maximum gap width (μm)	
*AirNivol*	160.2	250.80	763.65	
*ALL IN*	248.0	405.99	1380.29	
*Arc Angel*	402.3	805.06	2020.41	
*F22*	106.7	224.83	857.28	
*Invisalign*	180.6	269.23	915.86	
*Nuvola*	257.5	380.12	1342.15

### 2D analysis

**Fig. 7:**
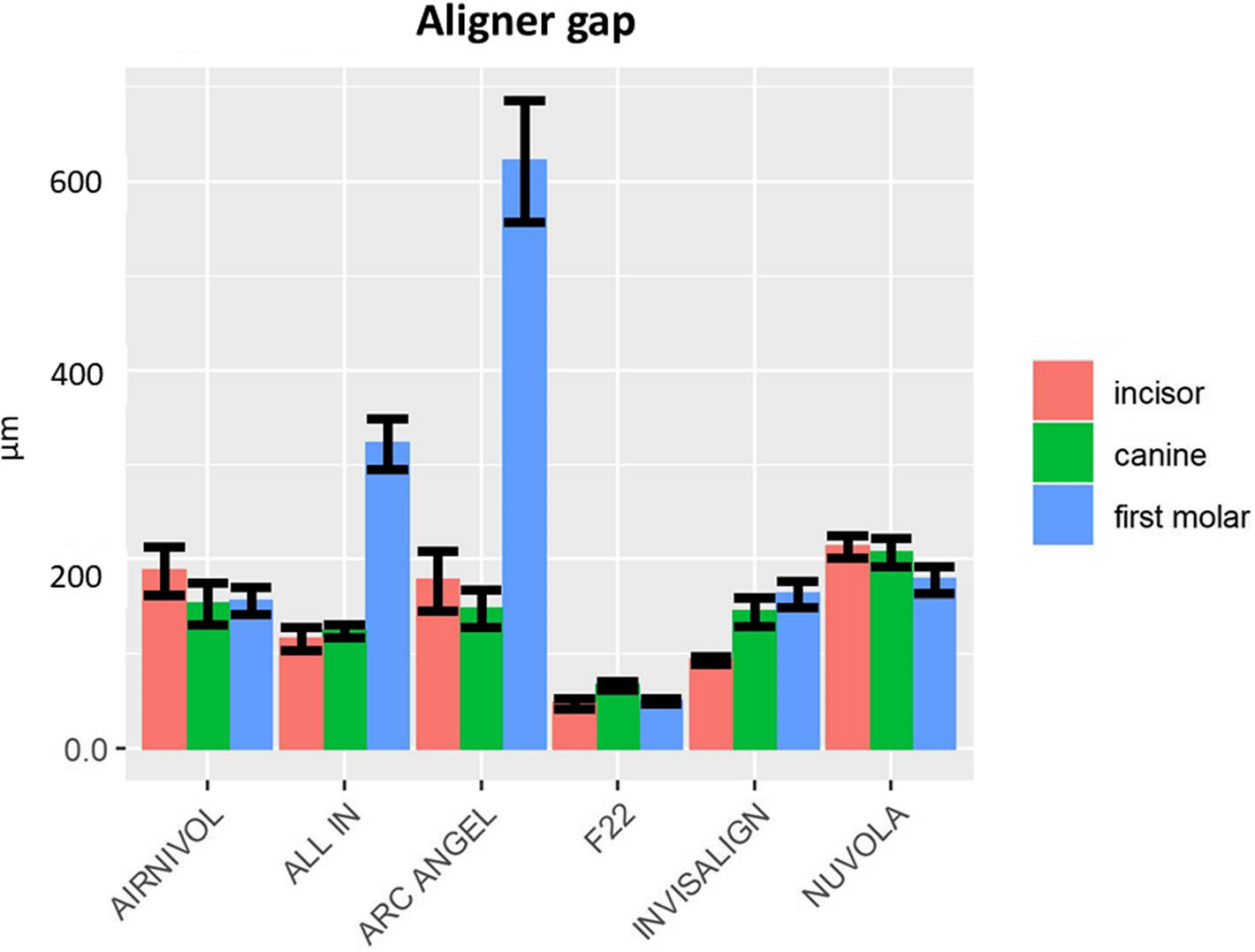
Graphical representation of aligner gap values and SD

**Fig. 8:**
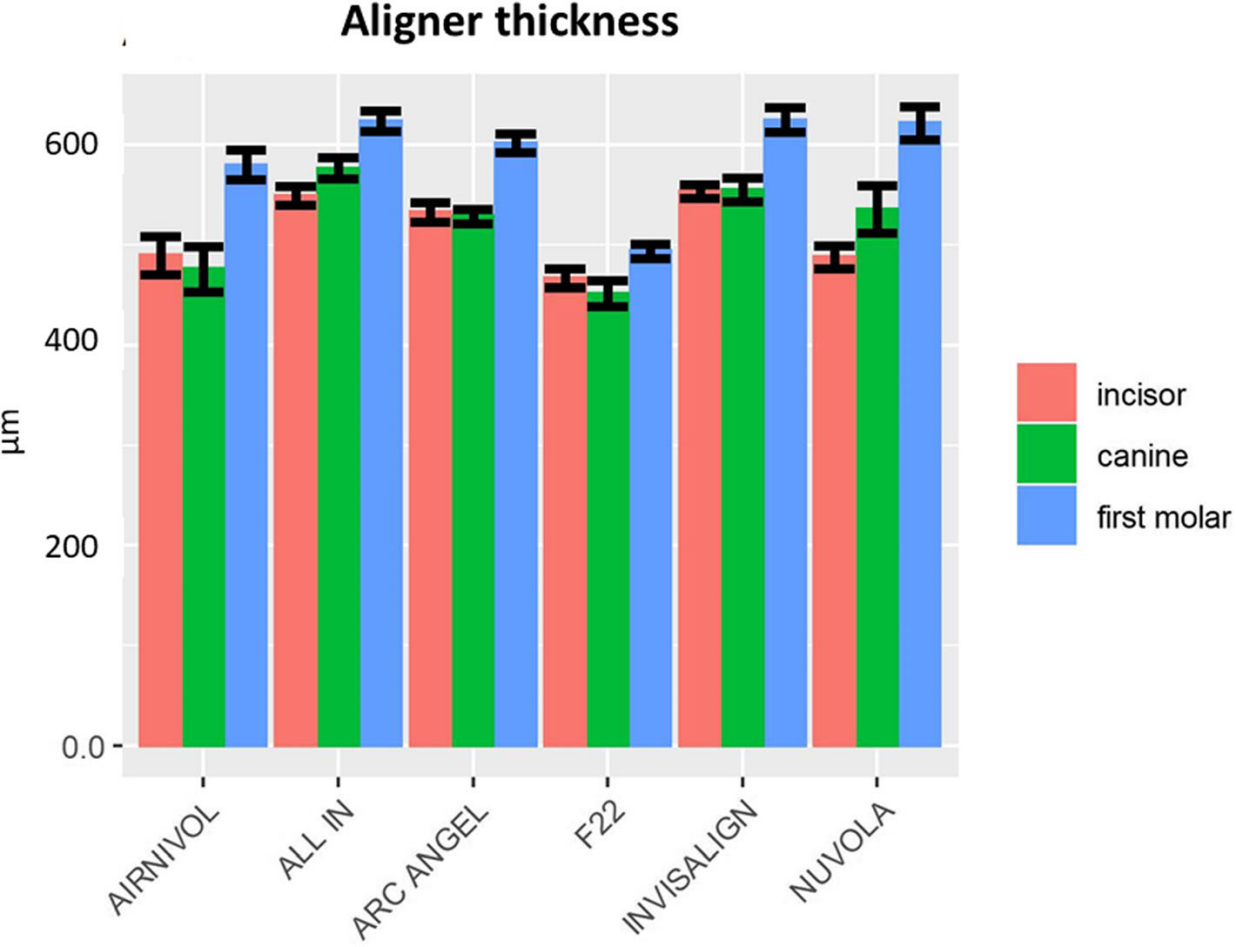
Graphical representation of aligner thickness values and SD

Table 3 shows the mean linear measurements (μm) and SD for both aligner gap and thickness for each investigated tooth, which are represented graphically in Figs. 7 and 8.

**Table 3:** Mean gap and aligner thickness values (μm) and their respective standard deviations (SD) of the six systems by tooth

		Tooth					
		Incisor		Canine		First molar	
		Mean (μm)	SD	Mean (μm)	SD	Mean (μm)	SD
Gap width	Air Nivol	187.7	127.22	152.82	107.83	156.03	76.6
	All In	115.81	61.98	124.19	35.46	322.58	147.21
	Arc Angel	177.28	157.49	147.89	99.95	621.47	353.41
	F22	47.4	26.73	66.04	22.56	49.54	15.36
	Invisalign	93.22	19.35	144.57	67.41	162.97	80.12
	Nuvola	213.14	59.78	207.01	74.34	178.31	84.51
Aligner thickness	Air Nivol	489.64	95.76	475.90	111.13	580.26	81.87
	All In	549.21	46.61	576.86	52.70	624.36	56.03
	Arc Angel	532.92	48.42	528.67	35.40	601.98	49.68
	F22	467.36	46.66	451.53	62.37	493.84	44.04
	Invisalign	553.4	29.75	555.37	52.06	625.23	73
	Nuvola	487.9	58.62	535.94	119.12	622.07	98.91

The two-way ANOVA showed significant differences between the systems in terms of both aligner thickness (*P* = 0.012) and aligner gap (*P* = 0). Tukey’s post hoc analysis (*P* < 0.05) revealed that, for the aligner gap (Table 4), the differences between the various aligner systems reached statistical significance for 4 pairwise

**Table 4:** Statistically significant comparisons for the aligner gap measure (*P*< 0.05*,*P*< 0.01**,*P*< 0.001***)

Aligner gap							
Tooth	Comparison	Estimate (μm)	SE (μm)	Df	Lower CL (μm)	Upper CL (μm)	*P*value
Canine	F22-Nuvola	− 140.97	34.27	467	− 239.04	− 42.90	*P* < 0.001***
Incisor	Air Nivol-F22	140.30	34.27	467	42.24	238.37	*P* < 0.001***
	Arc Angel-F22	129.88	34.27	467	31.81	227.94	*P* < 0.001***
	F22-Nuvola	− 165.74	34.27	467	− 263.81	− 67.67	*P* < 0.001***
	Invisalign-Nuvola	− 119.92	36.35	467	− 223.94	− 15.90	*P* < 0.01**
First molar	Air Nivol-ALL IN	− 166.56	31.28	467	− 256.08	− 77.04	*P* < 0.001***
	Air Nivol–Arc Angel	− 465.44	31.28	467	− 554.96	− 375.92	*P* < 0.001***
	Air Nivol-F22	106.48	30.15	467	20.21	192.75	*P* < 0.01**
	ALL IN-Arc Angel	− 298.88	31.28	467	− 388.41	− 209.36	*P* < 0.001***
	ALL IN-F22	273.04	30.15	467	186.77	359.31	*P* < 0.001***
	ALL IN-Invisalign	159.62	30.15	467	73.35	245.88	*P* < 0.001***
	ALL IN-Nuvola	144.27	30.15	467	58.00	230.54	*P* < 0.001***
	Arc Angel-F22	571.92	30.15	467	485.66	658.19	*P* < 0.001***
	Arc Angel-Invisalign	458.50	30.15	467	372.23	544.77	*P* < 0.001***
	Arc Angel-Nuvola	443.15	30.15	467	356.89	529.42	*P* < 0.001***
	F22-Invisalign	− 113.42	28.96	467	− 196.30	− 30.54	*P* < 0.001***
	F22-Nuvola	− 128.77	28.96	467	− 211.65	− 45.89	*P* < 0.001***

comparisons at the incisor, 1 pairwise comparison at the canine, and 12 pairwise comparisons at the first molar, while, when aligner thickness was considered, 7 pairwise comparisons at the incisor were found to be statistically significant, as were 8 pairwise comparisons at the canine and 5 pairwise comparisons at the first molar (Table 5).

**Table 5:** Statistically significant comparisons for the aligner thickness measure (*P*< 0.05*,*P*< 0.01**,*P*< 0.001***)

Aligner thickness							
Tooth	Comparison	Estimate (μm)	SE (μm)	Df	Lower CL (μm)	Upper CL (μm)	*P*value
Canine	Air Nivol-ALL IN	− 1100.96	19.83	467	− 1157.71	− 144.22	*P* < 0.001***
	Air Nivol-Invisalign	− 179.47	21.03	467	− 1139.66	− 119.29	*P* < 0.01**
	Air Nivol-Nuvola	− 160.04	19.83	467	− 1116.78	− 13.30	*P* < 0.05*
	ALL IN-Arc Angel	48.19	19.83	467	− 18.55	104.93	*P* < 0.05*
	ALL IN–F22	125.33	19.83	467	68.58	182.07	*P* < 0.001***
	Arc Angel-F22	77.14	19.83	467	20.39	133.88	*P* < 0.01**
	F22-Invisalign	− 1103.84	21.03	467	− 1164.02	− 143.65	*P* < 0.001***
	F22-Nuvola	− 184.40	19.83	467	− 1141.15	− 127.66	*P* < 0.001***
Incisor	Air Nivol-ALL IN	− 159.57	19.83	467	− 1116.31	− 12.82	*P* < 0.05*
	Air Nivol-Invisalign	− 163.75	21.03	467	− 1123.94	− 13.57	*P* < 0.05*
	ALL IN-F22	81.84	19.83	467	25.10	138.59	*P* < 0.001***
	ALL IN-Nuvola	61.30	19.83	467	4.56	118.05	*P* < 0.05*
	Arc Angel-F22	65.56	19.83	467	8.81	122.30	*P* < 0.05*
	F22-Invisalign	− 186.03	21.03	467	− 1146.22	− 125.84	*P* < 0.001***
	Invisalign-Nuvola	65.49	21.03	467	5.30	125.68	*P* < 0.05*
First molar	Air Nivol-F22	86.42	17.44	467	36.50	136.33	*P* < 0.001***
	ALL IN-F22	130.53	17.44	467	80.61	180.44	*P* < 0.001***
	Arc Angel-F22	108.14	17.44	467	58.23	158.06	*P* < 0.001***
	F22-Invisalign	− 1131.39	16.76	467	− 1179.35	− 183.43	*P* < 0.001***
	F22-Nuvola	− 1128.23	16.76	467	− 1176.19	− 180.27	*P* < 0.001***

The two-way ANOVA at level of 2D points showed statistically significant differences (*P* < 0.05) for both measurements. Subsequent Tukey’s post hoc testing revealed that the significance threshold (*P* < 0.05) was reached in almost all cases, with the exception of six pairwise comparisons for aligner gap and one pairwise comparison for aligner thickness, demonstrating considerable heterogeneity among aligner systems (Table 6).

**Table 6:** Comparisons of gap and aligner thickness of investigated systems that do not reach statistical significance (*P* < 0.05*)

Value	Tooth	2D Point	Comparison	Estimate (μm)	SE (μm)	f	Lower CL (μm)	Lower CL (μm)	*P* Value
**Aligner Gap**	Incisor	5	ALL IN - Invisalign	0.79	1.24	388	-2.75	4.33	0.99
	Incisor	2	Arc Angel - Nuvola	1.58	1.24	388	-1.96	5.12	0.80
	Incisor	4	Air Nivol - ALL IN	-3.16	1.24	388	-6.70	0.38	0.11
	Canine	1	ALL IN - F22	1.596	1.24	388	-1.94	5.14	0.79
	First Molar	6	Invisalign - Nuvola	3.16	1.24	388	-0.38	6.70	0.11
	First Molar	2	Invisalign - Nuvola	3.16	1.24	388	-5.12	1.96	0.80
**Aligner** **thickness**	Canine	Canine	Air Nivol- F22	0.16	0.11	388	-0.16	1.96	0.72

## Discussion

The degree to which planned orthodontic movements are effectively achieved depends on many variables, among which the gap between the tooth and aligner. The force exerted on a tooth by the aligner can be dissipated by the combined action of the air in this gap and the flexibility of the periodontal ligament, which enables a tooth to move up to roughly 0.04 mm before any biochemical phenomenon at the beginning of orthodontic tooth movement (OTM) occurs [26]. Hence, to obtain a clinically efficacious force, it is necessary that the contact between the internal surface of the aligner and the tooth crown be as close as possible. On the other hand, aligner thickness is correlated to forces and moments exerted.

Therefore, we set out to investigate both the gap and aligner thickness in six aligner systems, performing both 2D and 3D measurements. Data were obtained using nano-CT, an investigative method that does not cause even microscopic alterations in the sample, and should therefore provide more accurate data than cutting machine and SEM analysis [21, 22].

Our 3D analysis showed that F22 displayed the lowest measures in terms of both gap volume and mean aligner gap width, while the Airnivol showed the lowest maximum gap width overall. The 2D analysis, on the other hand, revealed the great heterogeneity between aligner systems in terms of how intimately they come into contact with the tooth surface, especially at the first molar. In essence, these results indicate that all aligner systems tested generally provide good fit in the anterior sectors, while to achieve predictable orthodontic movement and good anchorage in the posterior sectors, the choice needs to be more careful. Indeed, the anatomy of the occlusal sector is more complex in the posterior teeth, and the thermoforming process used to make the aligner allows less stretching in this sector. It is therefore conceivable that the observed differences in aligner behaviour may be due to the different temperature and pressure parameters adopted during manufacturing, as well as the viscosity and elasticity of the materials used [15, 21, 22].

Aligner thickness may also play a role in this regard, and, in fact, our 2D analysis revealed that the aligners were generally thicker in the posterior than the anterior sectors, although both revealed a reduced values of thickness respect to pre-thermoforming values. Nonetheless, pairwise comparison of the various system in terms of the points on the grid revealed, once again, considerable heterogeneity between aligners, with differences failing to reach statistical significance (*P* > 0.05) in only seven cases. In this case too, differences are likely ascribable to the manufacturing process and materials used to make the aligners.

Taking the gap and aligner thickness data as a whole, it is clear that aligners fit better in the anterior than the posterior sector, and this general trend leads us to believe that fit is influenced by tooth anatomy to a greater extent than by the differences in the thermoforming process or the physical characteristics of the aligner materials.

Authors have stated that differences in aligner thickness can account for different mechanical properties with differences in load-deflection curves [15]. Thinner aligners usually exhibit lower forces at the beginning, with a more progressive decrease of forces exerted during the following hours with respect to thicker aligners [20], thus providing more gentle and constant forces in the anterior region where extension of root surfaces is smaller.

Moreover, the augmented thickness of aligners in the posterior teeth reflects the rationale that posterior teeth, which have greater root surface extension with respect to anterior teeth [27], could benefit from higher forces and moments delivered by aligners. However, the greater gap we recorded in the posterior regions complicate this consideration.

The findings of this in vitro study raise some issues that clinicians should be aware of. Specifically, some aligner systems seem to provide more intimate contact with tooth surface with respect to others, and this could affect the efficiency of CAT. As a matter of fact, the aligner gap could be considered as similar to the archwire/bracket slot play in fixed appliances, and this is directly correlated to transmission of forces and moments necessary to obtain good OTM [27]. Therefore, it is conceivable that a better fitting aligner would more readily exert moments and forces, thanks to the intimate contact between tooth surface and the inner surface of aligner. Less dissipation of the initial information, especially during the first stage, due to a smaller aligner gap would make CAT more predictable.

With this in mind, it is interesting to note that some tested aligner brands displayed significantly greater values for aligner gap, especially at the posterior teeth, a major component of the common staging protocol for translational movement (0.25–0.33 mm) used by the vast majority of aligners [9]; therefore, it is important to be aware that transmission of initial forces may be rendered inefficient or null during the early steps with a poorly fitting aligner system, thereby delaying the beginning of OTM. Each aligner brand we investigated had the same nominal thickness before thermoforming procedures, but differences in the manufacture and the material of the aligners make it difficult to provide a meaningful comparison. Once manufactured, however, F22 aligners displayed the best overall fit, in both 3D (gap volume and mean gap width) and 2D analysis with respect to the other brands, but the clinical (efficiency of treatment and patient comfort) and mechanical consequences (force and moment exerted) of this finding requires further investigation. That being said, some aligners we tested did present gap values greater than 0.25–0.33 mm at the posterior teeth, meaning that the intimate contact linked to the transmission of orthodontic forces and moments during the common translational staging step may be delayed.

Our results also provide a springboard for further investigation into aligner thickness. The F22 yielded the lowest values for this parameter, which could partially explain the good optical properties it displayed in a previous report [15]; however, to what extent the small differences in thickness we found could improve the optical properties should be elucidated by further research, which is also warranted into the implications of the increased forces and moments exerted by thicker aligners.

As this was an in vitro study, we did not take into account the presence of saliva, which may cause the aligner to expand to a greater or lesser degree depending on its physical characteristics [23], or the effect of chewing, which is likely to deform an aligner and thereby affect both its thickness and the fit. Future studies should also investigate the interactions of different types of attachments and the aligner thickness and gap of a wide range of aligner systems, preferably using high-resolution micro-CT analysis, which is both non-invasive and precise.

## Conclusions

- The aligners tested generally present good fit (especially in anterior regions) and a reduced thickness with respect to the pre-thermoforming thickness.
- 3D analysis showed how F22 has the best overall fit (gap volume and mean gap width).
- 2D analysis showed differences for both measurements. Differences in aligner gap do exist, especially in the posterior sectors, while differences in aligner thickness appeared more evenly distributed among teeth investigated. Comparisons for both measurements at 2D points outlined a high heterogeneity among aligner systems.
- The differences between aligner systems that we reveal may affect their clinical efficacy and efficiency, and further investigation is warranted.

### Notes

Note that the image in Fig. 1 is not a volume rendering of the void existing between the retainer and the teeth; it represents the medial axis function, or skeletal function or skeleton, of the void phase. The skeleton is a descriptor of shape. The skeleton function extracts from image data the centerline of interconnected regions. This means that the skeleton is a synthetic way to represent the shape of a pattern and in each point of the skeleton spatial graph the distance to the nearest boundary is stored. As a result, the function graph cannot cover the entire original space and some areas of Fig. 1 are clear.

## Data Availability

Not applicable

## Abbreviations

CAT: Clear aligner therapy
CT: Computed tomography
